# Supplementary material for: Numerical Integration of Slater Basis Functions Over Prolate Spheroidal Grids
Source: J Comput Chem. 2026 Jan 5;47(1):e70291. doi: 10.1002/jcc.70291 (PMC12768307; doi:10.1002/jcc.70291)
Supplement: Supplementary file 1 — Data S1: The information contains basis set zeta values for all centers used in this paper, geometries for all systems, grid sizes used to generate Figures 3, 4, 5. Plot of N SP convergence for the nonequilibrium OF2 geometry. Plots of all basis combinations for 2‐center Coulomb integrals varying with displacement along 16 vectors. Plots that show extrapolation to FCI for Table 4 in GTO and STO basis sets. [file JCC-47-0-s001.docx]

**Supplemental Information**

**Numerical Integration of Slater Basis Functions Over Prolate Spheroidal Grids**

Alexander Stark^1^, Nathan Meier^1^, Jeffrey Hatch^1^, Joshua Kammeraad^1^, Duy-Khoi Dang^1^, Paul Zimmerman^1^

^1^Department of Chemistry, University of Michigan, Ann Arbor, Michigan, US.

*paulzim@umich.edu

Table of Contents

[I. Slater Basis Sets 2](#_Toc210997415)

[II. Geometries 6](#_Toc210997416)

[III. PS Grid Sizes 8](#_Toc210997417)

[IV. Figure S1 9](#_Toc210997418)

[V. Figure S2 10](#_Toc210997419)

[VI. Figures S3 and S4 12](#_Toc210997420)

#

# I. Slater Basis Sets

**Table S1:** Slater basis exponents for TZ, QZ, and 5Z basis functions applied to molecules in Figures 3, 4, 5, 7, and S1 and Tables 1, 2, 3 and 4.

| TZ | | QZ | | 5Z | |
| --- | --- | --- | --- | --- | --- |
| H | | | | | |
| 1s | 2.280 | 1s | 4.000 | 1s | 4.100 |
| 1s | 1.520 | 1s | 2.500 | 1s | 2.929 |
| 1s | 1.013 | 1s | 1.5625 | 1s | 2.092 |
| 2p | 2.100 | 1s | 0.977 | 1s | 1.494 |
| 2p | 1.500 | 2p | 3.200 | 1s | 1.067 |
|  |  | 2p | 2.286 | 2p | 4.000 |
|  |  | 2p | 1.633 | 2p | 2.353 |
|  |  | 3d | 2.200 | 2p | 1.384 |
|  |  |  |  | 2p | 0.814 |
|  |  |  |  | 3d | 3.400 |
|  |  |  |  | 3d | 1.700 |
| C | | | | | |
| 1s | 8.689 | 1s | 11.271 | 1s | 15.000 |
| 1s | 5.323 | 1s | 7.653 | 1s | 10.714 |
| 1s | 3.261 | 1s | 5.196 | 1s | 7.653 |
| 1s | 1.998 | 1s | 3.528 | 1s | 5.466 |
| 1s | 1.224 | 1s | 2.396 | 1s | 3.905 |
| 1s | 0.750 | 1s | 1.627 | 1s | 2.789 |
| 2p | 4.000 | 1s | 1.105 | 1s | 1.992 |
| 2p | 2.051 | 1s | 0.750 | 1s | 1.423 |
| 2p | 1.052 | 2p | 7.667 | 1s | 1.016 |
| 3d | 3.400 | 2p | 3.932 | 2p | 11.667 |
| 3d | 1.900 | 2p | 2.016 | 2p | 6.306 |
|  |  | 2p | 1.034 | 2p | 3.409 |
|  |  | 3d | 5.000 | 2p | 1.843 |
|  |  | 3d | 3.125 | 2p | 0.996 |
|  |  | 3d | 1.953 | 3d | 5.900 |
|  |  | 4f | 2.600 | 3d | 4.214 |
|  |  |  |  | 3d | 3.010 |
|  |  |  |  | 3d | 2.150 |
|  |  |  |  | 4f | 3.600 |
|  |  |  |  | 4f | 1.800 |
| N | | | | | |
| 1s | 10.820 | 1s | 14.453 | 1s | 12.393 |
| 1s | 6.345 | 1s | 9.471 | 1s | 8.728 |
| 1s | 3.720 | 1s | 6.206 | 1s | 6.147 |
| 1s | 2.181 | 1s | 4.067 | 1s | 4.329 |
| 1s | 1.279 | 1s | 2.665 | 1s | 3.049 |
| 1s | 0.750 | 1s | 1.747 | 1s | 2.147 |
| 2p | 4.400 | 1s | 1.145 | 1s | 1.512 |
| 2p | 2.588 | 1s | 0.750 | 1s | 1.065 |
| 2p | 1.522 | 2p | 6.533 | 1s | 0.750 |
| 3d | 4.000 | 2p | 3.960 | 2p | 4.933 |
| 3d | 2.000 | 2p | 2.400 | 2p | 3.524 |
|  |  | 2p | 1.454 | 2p | 2.517 |
|  |  | 3d | 5.500 | 2p | 1.798 |
|  |  | 3d | 3.235 | 2p | 1.284 |
|  |  | 3d | 1.903 | 3d | 5.800 |
|  |  | 4f | 2.200 | 3d | 4.143 |
|  |  |  |  | 3d | 2.959 |
|  |  |  |  | 3d | 2.114 |
|  |  |  |  | 4f | 4.200 |
|  |  |  |  | 4f | 2.100 |
| O | | | | | |
| 1s | 13.351 | 1s | 18.099 | 1s | 14.948 |
| 1s | 7.506 | 1s | 11.485 | 1s | 10.283 |
| 1s | 4.220 | 1s | 7.288 | 1s | 7.075 |
| 1s | 2.373 | 1s | 4.625 | 1s | 4.867 |
| 1s | 1.334 | 1s | 2.935 | 1s | 3.348 |
| 1s | 0.750 | 1s | 1.862 | 1s | 2.303 |
| 2p | 5.700 | 1s | 1.182 | 1s | 1.585 |
| 2p | 2.850 | 1s | 0.750 | 1s | 1.090 |
| 2p | 1.425 | 2p | 7.000 | 1s | 0.750 |
| 3d | 4.000 | 2p | 4.000 | 2p | 8.667 |
| 3d | 2.000 | 2p | 2.286 | 2p | 5.417 |
|  |  | 2p | 1.306 | 2p | 3.385 |
|  |  | 3d | 6.000 | 2p | 2.116 |
|  |  | 3d | 3.529 | 2p | 1.322 |
|  |  | 3d | 2.076 | 3d | 5.600 |
|  |  | 4f | 2.600 | 3d | 4.000 |
|  |  |  |  | 3d | 2.857 |
|  |  |  |  | 3d | 2.041 |
|  |  |  |  | 4f | 4.400 |
|  |  |  |  | 4f | 2.200 |
| F | | | | | |
| 1s | 15.520 | 1s | 21.908 | 1s | 17.972 |
| 1s | 8.466 | 1s | 13.528 | 1s | 12.082 |
| 1s | 4.619 | 1s | 8.354 | 1s | 8.123 |
| 1s | 2.520 | 1s | 5.158 | 1s | 5.461 |
| 1s | 1.375 | 1s | 3.185 | 1s | 3.671 |
| 1s | 0.750 | 1s | 1.967 | 1s | 2.468 |
| 2p | 5.667 | 1s | 1.215 | 1s | 1.659 |
| 2p | 3.063 | 1s | 0.750 | 1s | 1.116 |
| 2p | 1.656 | 2p | 5.667 | 1s | 0.750 |
| 3d | 4.000 | 2p | 3.542 | 2p | 7.000 |
| 3d | 2.000 | 2p | 2.214 | 2p | 4.828 |
|  |  | 2p | 1.383 | 2p | 3.329 |
|  |  | 3d | 5.000 | 2p | 2.296 |
|  |  | 3d | 3.226 | 2p | 1.584 |
|  |  | 3d | 2.081 | 3d | 6.500 |
|  |  | 4f | 2.300 | 3d | 4.333 |
|  |  |  |  | 3d | 2.889 |
|  |  |  |  | 3d | 1.926 |
|  |  |  |  | 4f | 5.400 |
|  |  |  |  | 4f | 2.700 |
| Si | | | | | |
| 1s | 19.317 | 1s | 31.922 | 1s | 31.922 |
| 1s | 12.870 | 1s | 22.699 | 1s | 22.699 |
| 1s | 8.575 | 1s | 16.140 | 1s | 16.140 |
| 1s | 5.713 | 1s | 11.477 | 1s | 11.477 |
| 1s | 3.806 | 1s | 8.161 | 1s | 8.161 |
| 1s | 2.536 | 1s | 5.803 | 1s | 5.803 |
| 1s | 1.690 | 1s | 4.126 | 1s | 4.126 |
| 1s | 1.126 | 1s | 2.934 | 1s | 2.934 |
| 1s | 0.750 | 1s | 2.086 | 1s | 2.086 |
| 2p | 11.333 | 1s | 1.483 | 1s | 1.483 |
| 2p | 6.869 | 1s | 1.055 | 1s | 1.055 |
| 2p | 4.163 | 1s | 0.750 | 1s | 0.750 |
| 2p | 2.523 | 2p | 10.667 | 2p | 13.600 |
| 2p | 1.529 | 2p | 7.619 | 2p | 9.714 |
| 2p | 0.927 | 2p | 5.442 | 2p | 6.939 |
| 3d | 3.400 | 2p | 3.887 | 2p | 4.956 |
| 3d | 2.000 | 2p | 2.777 | 2p | 3.540 |
|  |  | 2p | 1.983 | 2p | 2.529 |
|  |  | 2p | 1.417 | 2p | 1.806 |
|  |  | 2p | 1.012 | 2p | 1.290 |
|  |  | 3d | 3.500 | 2p | 0.922 |
|  |  | 3d | 2.500 | 2p | 0.658 |
|  |  | 3d | 1.786 | 3d | 5.400 |
|  |  | 4f | 2.200 | 3d | 3.857 |
|  |  |  |  | 3d | 2.755 |
|  |  |  |  | 3d | 1.968 |
|  |  |  |  | 4f | 4.400 |
|  |  |  |  | 4f | 2.200 |
| S | | | | | |
| 1s | 23.379 | 1s | 31.547 | 1s | 31.547 |
| 1s | 15.209 | 1s | 22.456 | 1s | 22.456 |
| 1s | 9.894 | 1s | 15.985 | 1s | 15.985 |
| 1s | 6.437 | 1s | 11.380 | 1s | 11.378 |
| 1s | 4.187 | 1s | 8.099 | 1s | 8.099 |
| 1s | 2.724 | 1s | 5.765 | 1s | 5.765 |
| 1s | 1.772 | 1s | 4.104 | 1s | 4.104 |
| 1s | 1.153 | 1s | 2.921 | 1s | 2.921 |
| 1s | 0.750 | 1s | 2.079 | 1s | 2.079 |
| 2p | 12.800 | 1s | 1.480 | 1s | 1.480 |
| 2p | 8.000 | 1s | 1.054 | 1s | 1.054 |
| 2p | 5.000 | 1s | 0.750 | 1s | 0.750 |
| 2p | 3.125 | 2p | 13.067 | 2p | 17.067 |
| 2p | 1.953 | 2p | 9.333 | 2p | 12.190 |
| 2p | 1.221 | 2p | 6.667 | 2p | 8.707 |
| 3d | 3.700 | 2p | 4.762 | 2p | 6.220 |
| 3d | 2.000 | 2p | 3.401 | 2p | 4.443 |
|  |  | 2p | 2.430 | 2p | 3.173 |
|  |  | 2p | 1.735 | 2p | 2.267 |
|  |  | 2p | 1.240 | 2p | 1.619 |
|  |  | 3d | 3.500 | 2p | 1.156 |
|  |  | 3d | 2.500 | 2p | 0.826 |
|  |  | 3d | 1.786 | 3d | 4.600 |
|  |  | 4f | 2.300 | 3d | 3.286 |
|  |  |  |  | 3d | 2.347 |
|  |  |  |  | 3d | 1.676 |
|  |  |  |  | 4f | 2.800 |
|  |  |  |  | 4f | 1.400 |
| Cl | | | | | |
| 1s | 21.825 | 1s | 31.403 | 1s | 31.747 |
| 1s | 15.156 | 1s | 22.363 | 1s | 22.585 |
| 1s | 10.525 | 1s | 15.925 | 1s | 16.068 |
| 1s | 7.309 | 1s | 11.341 | 1s | 11.431 |
| 1s | 5.076 | 1s | 8.076 | 1s | 8.132 |
| 1s | 3.525 | 1s | 5.751 | 1s | 5.785 |
| 1s | 2.448 | 1s | 4.095 | 1s | 4.116 |
| 1s | 1.700 | 1s | 2.916 | 1s | 2.928 |
| 1s | 1.180 | 1s | 2.077 | 1s | 2.083 |
| 2p | 22.000 | 1s | 1.479 | 1s | 1.482 |
| 2p | 12.571 | 1s | 1.053 | 1s | 1.054 |
| 2p | 7.184 | 1s | 0.750 | 1s | 0.750 |
| 2p | 4.105 | 2p | 14.667 | 2p | 18.600 |
| 2p | 2.346 | 2p | 10.476 | 2p | 13.286 |
| 2p | 1.340 | 2p | 7.483 | 2p | 9.490 |
| 3d | 3.7 | 2p | 5.345 | 2p | 6.778 |
| 3d | 2.0 | 2p | 3.818 | 2p | 4.842 |
|  |  | 2p | 2.727 | 2p | 3.458 |
|  |  | 2p | 1.948 | 2p | 2.470 |
|  |  | 2p | 1.391 | 2p | 1.764 |
|  |  | 3d | 4.000 | 2p | 1.260 |
|  |  | 3d | 2.857 | 2p | 0.900 |
|  |  | 3d | 2.041 | 3d | 5.100 |
|  |  | 4f | 2.200 | 3d | 3.643 |
|  |  |  |  | 3d | 2.602 |
|  |  |  |  | 3d | 1.859 |
|  |  |  |  | 4f | 2.400 |
|  |  |  |  | 4f | 1.200 |

# II. Geometries

**Table S2:** Geometries in Ångstrom for systems used in Figures 3, 4, 5, 7 and Tables 1, 2, and 3 as well as the large bond distance OF_2_ structure used to obtain Figure S1. Geometries with 3 or greater atoms were optimized using DFT with a ωB97X functional in a cc-pVQZ basis, unless specified elsewhere. The 1,3 propanediyl system was optimized with the B3LYP functional in a 6-31G* basis.

| Molecule | Atom | X | Y | Z |
| --- | --- | --- | --- | --- |
| HF |  |  |  |  |
|  | H | 0.0 | 0.0 | 0.0 |
|  | F | 0.0 | 0.0 | 0.9170 |
| C_2_ |  |  |  |  |
|  | C | 0.0 | 0.0 | 0.0 |
|  | C | 0.0 | 0.0 | 1.147 |
| N_2_ |  |  |  |  |
|  | N | 0.0 | 0.0 | 0.0 |
|  | N | 0.0 | 0.0 | 1.098 |
| CO |  |  |  |  |
|  | C | 0.0 | 0.0 | 0.0 |
|  | O | 0.0 | 0.0 | 1.128 |
| NO |  |  |  |  |
|  | N | 0.0 | 0.0 | 0.0 |
|  | O | 0.0 | 0.0 | 1.054 |
| O_2_ |  |  |  |  |
|  | O | 0.0 | 0.0 | 0.0 |
|  | O | 0.0 | 0.0 | 1.208 |
| HCl |  |  |  |  |
|  | H | 0.0 | 0.0 | 0.0 |
|  | Cl | 0.0 | 0.0 | 1.275 |
| CS |  |  |  |  |
|  | C | 0.0 | 0.0 | 0.0 |
|  | S | 0.0 | 0.0 | 1.535 |
| SiO |  |  |  |  |
|  | Si | 0.0 | 0.0 | 0.0 |
|  | O | 0.0 | 0.0 | 1.510 |
| ClF |  |  |  |  |
|  | Cl | 0.0 | 0.0 | 1.628 |
|  | F | 0.0 | 0.0 | 0.0 |
| Cl_2_ |  |  |  |  |
|  | Cl | 0.0 | 0.0 | 0.0 |
|  | Cl | 0.0 | 0.0 | 1.988 |
| H_2_O |  |  |  |  |
|  | H | 0.03662 | 0.2867 | -0.7420 |
|  | H | 0.006501 | 1.1120 | 0.5315 |
|  | O | -0.003118 | 0.2113 | 0.2105 |
| HCN |  |  |  |  |
|  | H | -0.008949 | 0.01992 | 1.092 |
|  | C | 0.01279 | 0.05594 | 0.02497 |
|  | N | 0.03616 | 0.09413 | -1.117 |
| OFH |  |  |  |  |
|  | O | -0.004260 | 0.02793 | 0.1246 |
|  | F | 0.005461 | 1.316 | 0.6854 |
|  | H | 0.03880 | 0.2657 | -0.8100 |
| N_3_ |  |  |  |  |
|  | N | 0.01331 | 0.5362 | 0.0003591 |
|  | N | -0.005540 | 1.255 | 0.9236 |
|  | N | 0.03223 | -0.1809 | -0.9239 |
| N_2_O |  |  |  |  |
|  | N | 0.03390 | -0.1616 | -1.002 |
|  | N | 0.01371 | 0.3602 | -0.01847 |
|  | O | -0.007608 | 0.9114 | 1.020 |
| NO_2_ |  |  |  |  |
|  | N | -0.008446 | -0.02460 | 0.1907 |
|  | O | 0.003103 | 1.029 | 0.8568 |
|  | O | 0.04534 | 0.1060 | -1.0475 |
| SFH |  |  |  |  |
|  | S | -0.01224 | -0.07200 | 0.2630 |
|  | F | 0.005247 | 1.462 | 0.7820 |
|  | H | 0.04700 | 0.2196 | -1.045 |
| OF_2_ |  |  |  |  |
|  | O | -0.01081 | 0.06083 | 0.3100 |
|  | F | 0.004032 | 1.367 | 0.7531 |
|  | F | 0.04678 | 0.1819 | -1.063 |
| SO_2_ |  |  |  |  |
|  | S | -0.01091 | -0.06565 | 0.2156 |
|  | O | 0.001496 | 1.134 | 0.9929 |
|  | O | 0.04941 | 0.04196 | -1.208 |
| CS_2_ |  |  |  |  |
|  | C | 0.01333 | 0.5366 | 0.00005083 |
|  | S | -0.01564 | 1.410 | 1.275 |
|  | S | 0.04231 | -0.3368 | -1.275 |
| OF_2_ (long bond) |  |  |  |  |
|  | O | 0.0 | 0.0 | 0.0 |
|  | F | 0.0 | 0.0 | 1.380 |
|  | F | 0.0 | 40.23 | -9.810 |
| 1,3 Propanediyl |  |  |  |  |
|  | C | 1.278 | 0.0 | 0.2597 |
|  | C | 0.0 | 0.0 | -0.5852 |
|  | C | -1.278 | 0.0 | 0.2597 |
|  | H | 1.325 | -0.8846 | 0.9073 |
|  | H | 1.325 | 0.8846 | 0.9073 |
|  | H | 0.0 | 0.8776 | -1.246 |
|  | H | 0.0 | -0.8776 | -1.246 |
|  | H | -1.325 | -0.8846 | 0.9073 |
|  | H | -1.325 | 0.8846 | 0.9073 |

# III. PS Grid Sizes

**Table S3:** Grid sizes used for Figures 3 and 4 (left) and Figure 5 (right). All combinations of radial and angular number of points were considered (left). Each row of values represents parameters for a single calculation in the generation of Figure 5 (right).

| $N_{\mu}$ | $N_{\nu}$ | $N_{\phi}$ |  | $N_{\mu}$ | $N_{\nu}$ | $N_{\phi}$ |
| --- | --- | --- | --- | --- | --- | --- |
| 26 | 20 | 8 |  | 13 | 20 | 8 |
|  |  |  |  | 14 | 20 | 8 |
| 27 | 22 | 9 |  | 15 | 22 | 9 |
|  |  |  |  | 16 | 22 | 9 |
| 28 | 24 | 10 |  | 17 | 24 | 10 |
|  |  |  |  | 18 | 24 | 10 |
| 29 | 26 | 11 |  | 19 | 26 | 11 |
|  |  |  |  | 20 | 26 | 11 |
| 30 | 28 | 12 |  | 21 | 28 | 12 |
|  |  |  |  | 22 | 28 | 12 |
| 31 | 30 | 13 |  | 23 | 30 | 13 |
|  |  |  |  | 24 | 30 | 13 |
| 32 | 32 | 14 |  | 25 | 32 | 14 |
|  |  |  |  | 26 | 32 | 14 |
| 33 | 34 | 15 |  | 27 | 34 | 15 |
|  |  |  |  | 28 | 34 | 15 |
| 34 | 36 | 16 |  | 29 | 36 | 16 |
|  |  |  |  | 30 | 36 | 16 |
| 35 | 38 | 17 |  | 31 | 38 | 17 |
|  |  |  |  | 32 | 38 | 17 |
| 36 | 40 | 18 |  | 33 | 40 | 18 |
|  |  |  |  | 34 | 40 | 18 |
| 37 | 42 | 19 |  | 35 | 42 | 19 |
|  |  |  |  | 36 | 42 | 19 |
| 38 | 44 | 20 |  | 37 | 44 | 20 |
|  |  |  |  | 38 | 44 | 20 |

# IV. Figure S1


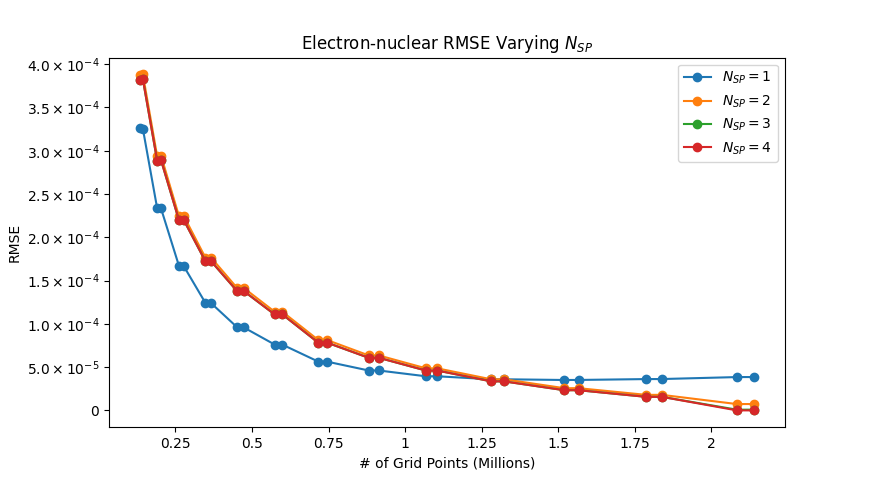


**Figure S1:** Comparison of the RMSE (Ha) of the electron-nuclear attraction elements for different divisions of the third center within the PS integration grid. Calculations performed on the OF_2_ system with one OF bond at a 41.41Å distance.

The RMSE of the electron-nuclear attraction integral for OF_2_ with an OF bond at ~30 times equilibrium bond length confirms the error is reduced at large grid discretization up to $N_{SP}=3$. At smaller grid sizes when $N_{SP}>1$ the RMSE of the electron-nuclear attraction integral increases, this phenomenon is believed to be caused by a cancellation of error when the grid around the third center is coarser.

# V. Figure S2


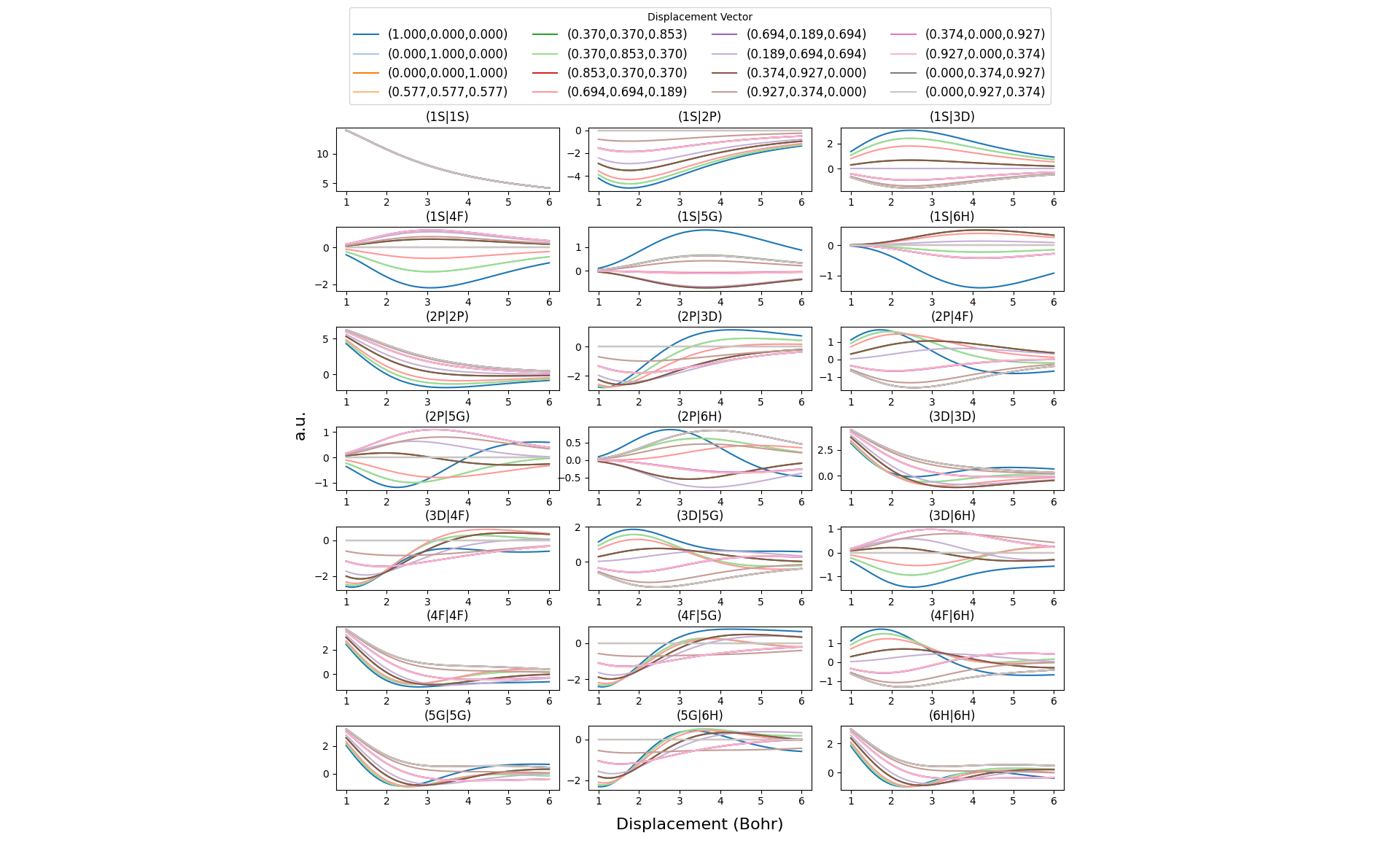


**Figure S2:** All combinations of 2-center Coulomb integrals plotted along 16 vector directions^15^ as the two centers are separated. The legend indicates unit vectors for all 16 directions tested, all basis functions used have the exponent $\zeta=1$, and all basis functions were chosen to have $m=0$.

# VI. Figures S3 and S4

**
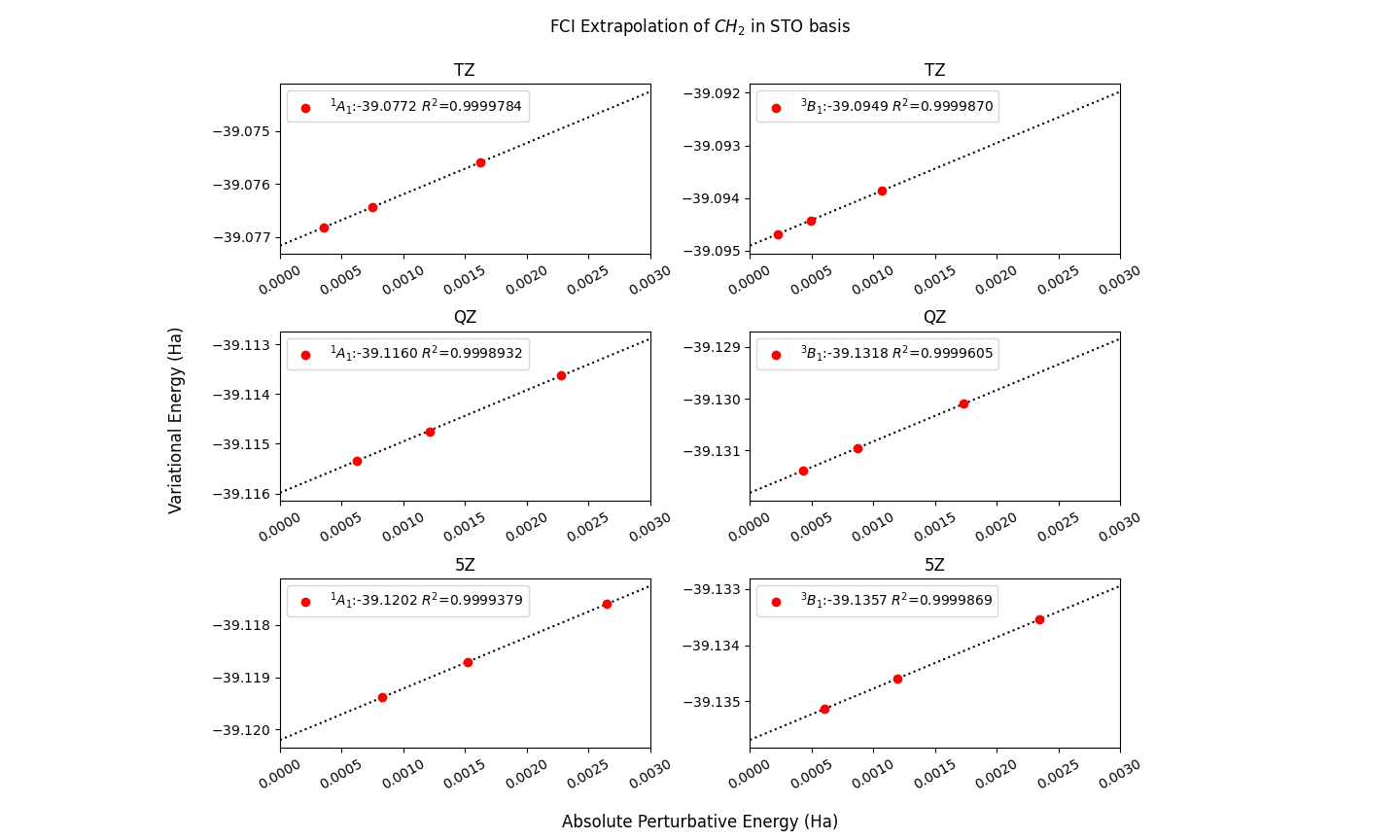
**

**Figure S3:** FCI extrapolation energies for methylene in the TZ, QZ, and 5Z basis for both the ^1^A_1_ and ^3^B_1_ states using STOs.

**
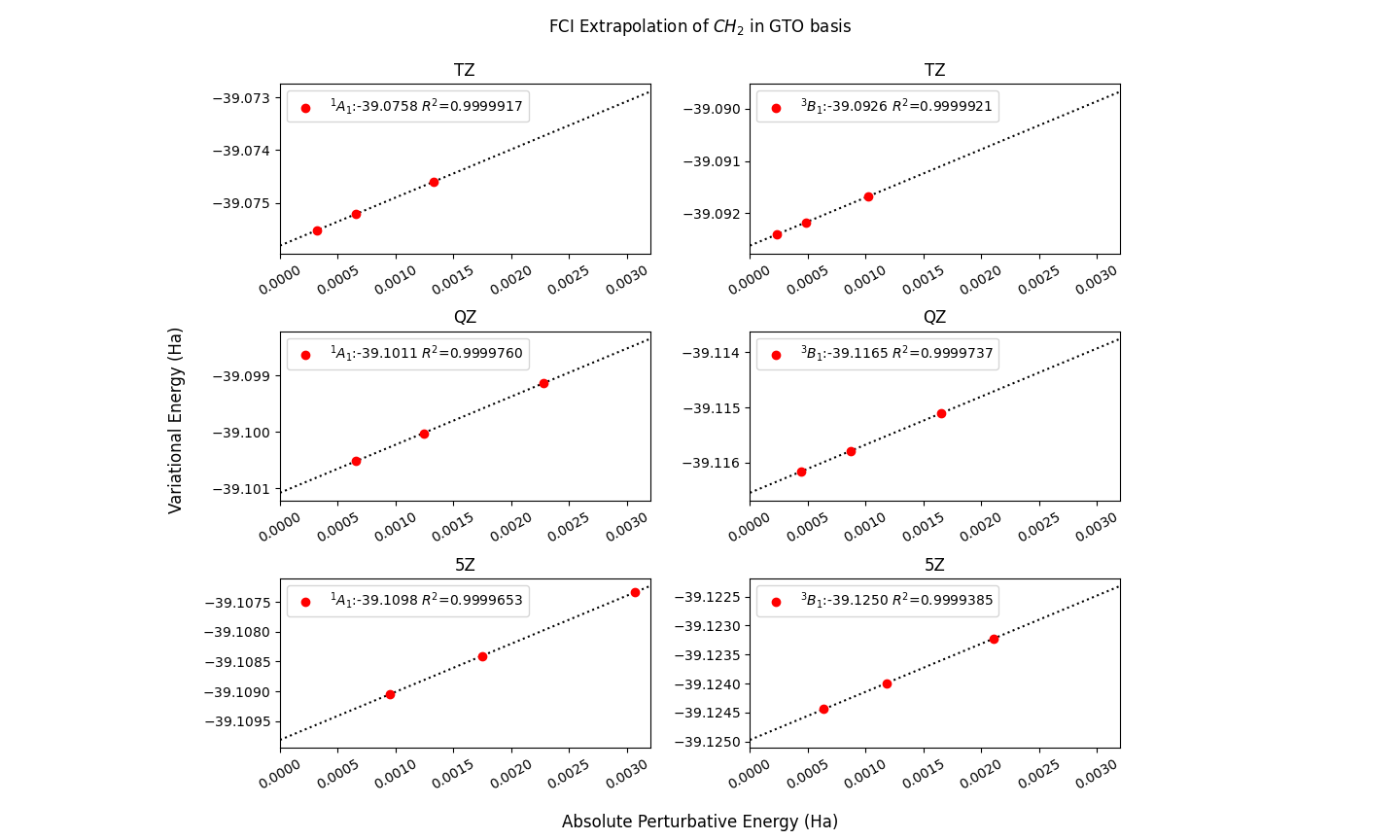
**

**Figure S4:** FCI extrapolation energies for methylene in the cc-pVTZ (TZ), cc-pVQZ (QZ), and cc-pV5Z (5Z) basis for both the ^1^A_1_ and ^3^B_1_ states using GTOs.

To obtain singlet-triplet gap energies for methylene with basis XZ (where X = T, Q, 5) three calculations are performed with varying $\varepsilon_{1}$ values ($\varepsilon_{1}=2\times{10}^{-4},1\times{10}^{-4},0.5\times{10}^{-4}$ Ha and $\varepsilon_{2}=5\times{10}^{-7}$ Ha) for the singlet and the triplet systems. The energies obtained from the extrapolations are then subtracted to find the singlet-triplet gap.
